# Identifying best practice for the supervision of mental health and psychosocial support in humanitarian emergencies: a Delphi study

**DOI:** 10.1186/s13033-022-00515-0

**Published:** 2022-02-07

**Authors:** Áine Travers, Nadeen Abujaber, Kelly A McBride, Pia Tingsted Blum, Nana Wiedemann, Frédérique Vallières

**Affiliations:** 1 Trinity Centre for Global Health (TCGH), School of Psychology, Trinity College Dublin, Dublin 2, College Green, Ireland; 2 Reference Centre for Psychosocial Support, International Federation of Red Cross and Red Crescent Societies (IFRC), Blegdamsvej 27, 2100 Copenhagen, Denmark

**Keywords:** Mental health and psychosocial support, MHPSS, Humanitarian emergency, Delphi

## Abstract

**Background:**

Supportive supervision has been shown to improve worker resilience and wellbeing, which are particularly important in the context of humanitarian emergency settings. Despite its noted importance however, supervision remains an under-prioritised area in mental health and psychosocial support (MHPSS).

**Method:**

The present study used a Delphi consensus-building methodology to examine levels of agreement among a diverse sample of MHPSS stakeholders (*n* = 48) on key ideas and concepts relating to supervision in humanitarian settings.

**Results:**

The majority of statements presented showed a high degree of consensus, with some receiving almost universal agreement, such as the importance of using active listening skills in the supervisory context and the need for supervisors to have access to their own supervisory support. However, disagreement on several points remained. For example, participants disagreed about whether the qualities required to be an effective supervisor can be taught, or whether they are more innate and should be screened for when recruiting supervisors. Gender differences in responses were also analysed, with potential associations between gender and level of agreement emerging in relation to statements about power dynamics, remote supervision, and intervention quality enhancement.

**Conclusions:**

The findings of the present study are discussed in terms of their implications for a forthcoming set of guidelines for supervision of MHPSS in humanitarian settings: The Integrated Model for Supervision (IMS).

## Introduction

Populations affected by humanitarian emergencies are at risk of adverse mental health outcomes relating to trauma exposure and ongoing adversity and challenging conditions in the crisis aftermath [6]. To mitigate this risk, mental health and psychosocial support (MHPSS) services—as a composite term that refers to “any type of local or outside support that aims to protect or promote psychosocial well-being and/or prevent or treat mental disorder” [11] - are commonly delivered, at varying degrees of intensity, to help affected populations to resume their lives and to cope with the mental health impacts of experiencing a humanitarian emergency. However, unified guidance relating to the supervision of MHPSS providers engaging in activities and interventions in emergency settings is currently lacking. This is particularly problematic given the unique challenges and stressors that face workers operating in humanitarian settings (e.g. Brooks et al. [4]; Aldamman et al. [2]), and the resulting elevated risk of adverse mental health outcomes and role-related stress reactions (e.g. Connorton et al. [7]; Strohmeier et al. [18]). For example, operating in complex and stressful environments to provide support to those who have experienced significant adversity increases workers’ vulnerability to vicarious trauma, secondary trauma and burnout [1, 5, 19]. This vulnerability is further compounded in situations where MHPSS workers are themselves from the communities affected by the humanitarian crisis, since prior exposure to traumatic events is a significant risk factor for the onset of occupational stress responses [10]. Indeed, several studies have shown that national (i.e., local) staff are at higher risk of adverse mental health outcomes compared to expatriate (i.e., international) staff, and that volunteers have worse outcomes than professional staff [2, 4, 7]. As well as placing the health and wellbeing of MHPSS workers at risk, these occupational stress-related problems create a barrier to worker retention and thereby threaten the sustainability and overall quality of services.

Supportive supervision is an approach to supervision that is collaborative and empowering, rather than controlling or fault-finding [26]. Supportive supervision may have potential to mitigate the impact of occupational stressors among humanitarian emergency workers by addressing risk factors such as a lack of role clarity, poor relationships with managers, and insufficient training and preparedness [4]. MHPSS in humanitarian emergencies is often delivered through task-shifting, or the rational redistribution of tasks from more highly qualified professionals to workers with fewer qualifications, to maximise efficiency in the delivery of services [25]. The availability of supportive supervision within task-shifting or task-sharing is therefore important to ensure that less qualified workers are adequately supported to deliver effective services. There is growing recognition that supportive supervision should be integrated into regular practice for all health professionals to promote worker wellbeing and health system sustainability [14] and it is recommended by the WHO as the preferred type of supervision for task-shifting frameworks [25].

Despite this acknowledgement of the importance of supportive supervision, supervision is often under-prioritised in MHPSS programming [13, 15]. Additionally, research in the field of MHPSS can be disconnected from practice, in that common and widely used practices tend to be under-researched, and more rigorously studied interventions are uncommonly used in humanitarian settings [23]. Finally, research has suggested that experiences and perceptions of humanitarian workers appear to vary considerably as a function of gender [20]. Indeed, gendered hierarchies common to MHPSS task-shifting frameworks have been noted, whereby supervising specialists such as psychiatrists tend to be mostly men, while less specialised workers are more likely to be women [13]. However, gender dimensions of humanitarian work in general is a highly under-researched area (e.g. Strohmeier and Panter‐Brick [20]).

The present study therefore aims to contribute towards bridging research-to-practice gaps in relation to supervision practice for MHPSS by generating an agreed upon set of ideas and principles for best practice guidance to be included in a new set of guidelines for the supervision of MHPSS in humanitarian emergencies, entitled the Integrated Model for Supervision (IMS). To achieve this aim, the current study had two objectives: (1) to determine (a) the level of consensus or disagreement on and (b) perceptions of a series of key statements for best practice guidance on supportive supervision in humanitarian contexts among a broad section of MHPSS actors and (2) to determine whether consensus scores vary as a function of gender.

## Method

### Study design

The Delphi method was used to gather stakeholder views on a set of twenty-eight statements relating to the supervision of MHPSS supervision in humanitarian emergencies. The Delphi method was chosen as a recognised method for building consensus among stakeholders, inviting experts to consider a set of key ideas or content, usually for the development of new measures or tools (Barratt and Heale [3]). The same set of statements are presented to experts in two or more rounds, with the aim of reducing the range of responses in each successive round; through consideration of the responses of others, the participants should eventually arrive at a point closer to agreement than where they began [9].

The present Delphi study included both quantitative and qualitative elements. Participants were invited to vote on the validity of the statements on a Likert response scale ranging from 1 (strongly disagree) to 5 (strongly agree), as well as to comment in support of their votes. The statements were developed through previous phases of research and consultations as part of the ongoing development of the IMS: these phases included (1) a desk review of literature relating to the supervision of MHPSS in humanitarian settings (McBride et al. [15]), (2) a series of key informant interviews (Perera et al. [17]) and (3) workshop discussions on best practice in supervision for MHPSS in humanitarian settings [12]. The statements were devised by the first author, consulting the cited documentation of the previous phases of consultation, with particular focus on identifying areas of disagreement or gaps in information from the process up to that point. The preliminary list consisted of fourteen statements, which were presented to the wider research team for discussion and expansion. Thirteen new statements were proposed by the research team and one statement was deleted, leaving a list of twenty-six. The new list was checked against project documentation by the first author and amended accordingly, and the list was then circulated to the research team for a second review. The second review resulted in one of the twenty-six statements being broken down into three components, resulting in the final list of twenty-eight statements. The list of statements was reviewed a final time for consistency and accessibility of language to produce the twenty-eight statements which were eventually presented to participants.

## Procedure

This study was conducted as part of the Supervision—The Missing Link project, which emphasises the use of participatory approaches for the co-development of a new unified set of guidelines (i.e., the IMS) for the supervision of MHPSS interventions in humanitarian settings. Participants were selected purposively via opt-in responses to a link posted on social media platforms of the IFRC as well as the newsletter of the IASC Reference Group on MHPSS in humanitarian settings. Participants from prior consultations and activities of the Supervision—The Missing Link project, and members of the project advisory group were also invited to participate. All individuals engaged in any type of MHPSS practice or research, both as supervisors and supervisees, were eligible to take part.

Participants who indicated willingness to take part were sent participant information leaflets. Having reviewed the information about the study, those who asked to receive the participation link were then directed to an electronic consent form on the specialised online platform for Delphi surveys, Welphi (www.welphi.com). The participation link was emailed to all participants on the same date, and they were given a two-week window to complete the survey.

Participants were presented with 28 statements related to supervision practices in emergency contexts. Participants were first asked to provide some basic demographic information (gender, age, occupation, nationality, supervisory experience) and were then asked to rate each statement using Likert response scale of: “strongly disagree”, “disagree”, “neither agree or disagree”, “agree”, “strongly agree” or “don’t know”. In addition, participants were encouraged to comment in support of their selection for each statement to capture their reflections on the validity of the statements.

After the two-week voting window, the first round was closed. Statements were adjusted after this first round if (1) consensus on a particular statement was not achieved and (2) the qualitative data from participant commentary provided clear direction for how to modify the statement. Following these adjustments, participants received a new link to participate in a second round, where the process of voting and commenting on the statements was repeated. In this second round however, participants could view the anonymous votes and comments of the other participants from the previous round and decide whether to revise their opinions or to stick with their previous vote(s).

## Participants

A total of *n* = 72 individuals initially agreed to participate. Of these, *n* = 48 participated in the first round. Only those who had participated in the first round could join the second round. A total of *n* = 37 participants completed both rounds. This represents an overall retention rate of 51%.

Of the sample who provided data for the first round (*n* = 48), 63% (*n* = 30) self-identified as female, 35% (*n* = 17) identified as male. The mean age was 42.11 years (SD = 8.97, range 26-69). In terms of experience with supervision, the majority (65%, *n* = 31) had experience of both providing and receiving supervision, while 4% (*n* = 2) only had experience of supervising, and a further 4% (*n* = 2) had experience only of being supervised. The remaining 27% (*n* = 13) provided no data on experiences of supervision. In terms of regions of nationality, the most represented region was Europe (*n* = 16), followed by Africa (*n* = 8), North America (n = 7), Asia (*n* = 5), South America (*n* = 2) and Australia (*n* = 1). The remainder of the sample did not provide data on their nationalities. Countries of origin listed were Australia, Bangladesh, Belgium, Bolivia, Bosnia and Herzegovina, Costa Rica, Croatia, Ecuador, Egypt, Ethiopia, Finland, France, Germany, Iceland, Ireland, Italy, Jordan, Kenya, Lebanon, Liberia, Malaysia, the Netherlands, Nigeria, Sweden, Spain, Uganda and the USA.

### Data analysis

To address objective 1a, consensus was defined as 75% who voted ‘agree’ or ‘strongly agree’ to a given item. The 75% cut-off was selected as the conventional cut-off point for Delphi studies (Diamond et al. [8]). The results present the final consensus scores from all 28 statements, as well as pertinent qualitative data submitted by participants in support of their choices, in fulfilment of objective 1b.

Bivariate analyses (t-tests) were used to address objective 2, assessing potential associations between gender and participants’ responses to the presented statements.

### Ethical considerations

Ethical approval for this study was obtained from the Centre for Health Policy and Management/Centre for Global Health Research Ethics Committee of Trinity College Dublin. All participant responses were de-identified, including when participants were able to view the responses of others, to preserve confidentiality.

## Results

### Consensus scores

The final consensus score (i.e., the percentage of participants who selected either ‘agree’ or ‘strongly agree’ for each statement) and the results of the bivariate analyses of gender differences in response patterns across all 28 statements are presented in Table 1. There was a high degree of consensus on most of the statements presented, with most statements passing the 75% threshold in the first round. Some statements achieved universal or near-universal agreement. For example, the statement, “Active listening is an important supervisory skill. This is demonstrated through (1) non-verbal signals, e.g. making eye contact, nodding or leaning forward to show that you are engaged, and (2) verbal practices, e.g. reflecting back or summarising what someone has told you, to check that you have understood it correctly” achieved 100% consensus. Similarly, the statement that read “Supervision works best if there are multiple tiers of supervision. For example, supervisors should also have access to supervision themselves,” achieved a final consensus score of 98%.

**Table 1:** Final consensus scores and bivariate analyses of gender differences in responses

	Final consensus score	Female M (SD)	Male M (SD)	t-values	*p*
**Active listening is an important supervisory skill. This is demonstrated through (1) non-verbal signals, e.g. making eye contact, nodding or leaning forward to show that you are engaged, and (2) verbal practices, e.g. reflecting back or summarising what someone has told you, to check that you have understood it correctly.**	**100%**	**4.92 (0.28)**	**4.64 (0.51)**	**t (12) =1.75**	**0.10**
**Supervision works best if there are multiple tiers of supervision. For example, supervisors should also have access to supervision themselves.**	**98%**	**4.56 (0.58)**	**4.45 (0.52)**	**t (34) = 0.52**	**0.61**
**Socratic questioning is a style of questioning that encourages logical reasoning, e.g. “If this is the case, then what will happen?” This type of questioning is a useful way for supervisors to encourage supervisees to use their knowledge to find solutions to problems independently.**	**97%**	**4.48 (0.51)**	**4.64 (0.51)**	**t (34) = -0.85**	**0.40**
**Supervision in the field of MHPSS and protection should include a focus on teaching or coaching the supervisee in specific skills.**	**95%**	**4.54 (0.76)**	**4.73 (0.47)**	**t (35) = −0.76**	**0.45**
**Interpreters who engage in supervision sessions should also have access to their own supervision and supports to help them manage difficult material discussed.**	**95%**	**4.48 (0.65)**	**4.55 (0.52)**	**t (34) = -0.29**	**0.77**
**Supervision is an essential component of any MHPSS training.**	**94%**	**4.60 (0.87)**	**4.82 (0.41)**	**t (34) = -0.79**	**0.43**
**Building supervisee’s confidence and sense of professional self-esteem is a core goal of supervision.**	**92%**	**4.44 (0.82)**	**4.36 (0.51)**	**t (34) = 0.29**	**0.78**
**Where the supervisor is also the supervisee’s manager, it is important that the supervisor clearly differentiates the boundaries of the two roles for the supervisee. For example, managerial and supervisory meetings should be held separately so that the two functions do not become confused.**	**92%**	**4.60 (0.65)**	**4.82 (0.41)**	**t (29) = −1.23**	**0.23**
**Supervision in the field of MHPSS and protection should provide emotional support to the supervisee.**	**91%**	**4.60 (0.58)**	**4.27 (0.79)**	**t (34) = 1.40**	**0.17**
**The ideal group size for group supervision is no more than 5 -6 supervisees.**	**90%**	**4.12 (0.67)**	**3.91 (0.83)**	**t (34) = 0.81**	**0.42**
**It is important for interpreters in MHPSS and protection interventions to sign a non-disclosure agreement to protect client confidentiality.**	**90%**	**4.56 (0.92)**	**4.45 (0.82)**	**t (34) = 0.33**	**0.75**
**Supervision in the field of MHPSS and protection should include some means of checking intervention fidelity.**	**90%**	**4.23 (0.86)**	**4.27 (0.65)**	**t (35) = −0.15**	**0.89**
**The best way for a supervisee to prepare for a supervision session is to reflect on what they find to be most challenging in their work, including specific cases, how they are managing stress in their work, and what they feel is going well.**	**89%**	**4.32 (0.63)**	**4.09 (0.54)**	**t (34) = 1.05**	**0.30**
**The ‘gold standard’ in supervision (i.e. the best possible approach) is: individualised supervision, delivered face-to-face by an external supervisor, who is not the supervisee’s manager. This should be available at all stages of providers’ professional practice. Sessions should be scheduled regularly (e.g. weekly, biweekly or monthly) or as frequently as required by the supervisee. Complementary approaches may be used in combination with individual supervision, but not as a replacement e.g. peer support or group sessions to facilitate multi-disciplinary case presentations.**	**89%**	**4.40 (0.87)**	**4.09 (0.94)**	**t (34) = 0.96**	**0.34**
**Good supervision should always involve goal-setting combined with focused feedback, and work towards these goals should be documented within sessions to enable supervisees to track their progress over time**	**87%**	**4.12 (1.01)**	**4.36 (0.67)**	**t (34) = −0.73**	**0.47**
**Supervisors should leave space at the end of every supervisory session to allow for discussion of what is working well in supervision, and what may be working less well.**	**86%**	**4.32 (0.95)**	**4.36 (0.92)**	**t (34) = −0.13**	**0.90**
**The issue of power dynamics between supervisor and supervisee is best addressed directly in supervision, by openly discussing sources of power and privilege that may be held by the supervisor and/or supervisee and how these might influence the supervisory relationship.**	**86%**	**4.00 (0.76)**	**4.55 (0.52)**	**t (34) = -2.15**	0.04*
**A supervision contract is the best way to ensure that the organization, supervisor, and supervisee are all in agreement about each of their roles within supervision and the nature, duration, and focus of the supervision relationship.**	**84%**	**4.36 (0.57)**	**3.82 (0.98)**	**t (13) = 1.71**	**0.11**
**Gender is essential to consider when pairing supervisors with supervisees, and gender compositions of supervisors-supervisee pairs should be considered on a context-by-context basis.**	**84%**	**4.36 (0.70)**	**4.00 (0.78)**	**t (34) = 1.38**	**0.18**
**In emergency contexts, it is essential to make concessions on how supervision sessions are conducted, but not on core aspects such as confidentiality.**	**82%**	**4.00 (1.26)**	**4.36 (0.51)**	**t (34) = −0.92**	**0.36**
**Supervisees should be able to discuss anything that affects their work during supervision sessions—even personal matters.**	**76%**	**3.84 (0.85)**	**4.27 (0.79)**	**t (34) = −1.44**	**0.16**
The skills needed to be a good supervisor, such as empathy, unconditional positive regard, and being a good active listener can be developed through training.	73%	3.96 (0.75)	4.09 (0.83)	t (33) = −0.47	0.64
With some additional effort, it is possible for remote supervision to be as successful as face-to-face supervision	68%	3.42 (1.06)	4.09 (0.70)	t (28) = -2.23	0.03*
The ‘sandwich approach’ (i.e. providing positive reinforcement before and after providing constructive criticism) is the best way for supervisors to deliver feedback.	67%	4.00 (0.76)	3.70 (1.06)	t (33) = 0.94	0.36
Peer supervision should only take place between experienced professionals to avoid the risk of misinformation being communicated	57%	3.30 (1.33)	3.82 (0.98)	t (32) = -1.14	0.26
Monitoring and evaluation tools, such as feedback forms, are the best way to ensure quality supervision in MHPSS and protection programming and supervisee progress.	51%	3.72 (0.98)	3.91 (1.14)	t (34) = −0.51	0.62
A key role of the supervisor is to guide supervisees’ discovery in relation to how to enhance the quality of MHPSS service delivery.	51%	3.38 (1.17)	4.18 (0.75)	t (29) = −2.45	0.02*
Supervision can only be considered supervision when taking place in specially designated spaces, such as an office, meeting room or other private space.	16%	2.50 (1.14)	2.45 (1.13)	t (33) = 0.11	0.91

Items that reached consensus are highlighted in bold. * denotes significance at alpha level of 0.05

A few statements did not reach the threshold in the first round but did pass it in the second round. Statement five, “Supervisees should be able to discuss anything that affects their work during supervision sessions—even personal matters” achieved a score of 69%, which rose to 76% in the second round. The reservations expressed by the participants in relation to this statement mainly pertained to concerns about blurring boundaries between supervision and personal therapy. As articulated by one participant:

*I consider this element in supervision as a “gray zone” between supervision and personal therapy. The important aspect is not crossing boundaries...*

However, most agreed that personal issues can at least be mentioned in supervision, but that the distinction between supervision and therapy is that supervision focuses mainly on how the issues affect the supervisee’s work. Several participants also highlighted the importance of the availability of external supports to allow supervisees to comprehensively address these issues.

…*an in-depth exploration of personal issues may not be appropriate in the context of the professional relationship. Even so, I would see it as a positive if [a personal issue] is disclosed and encourage discussions of how the supervisee can be supported or seek other support*.

Similarly, the statement “Supervisors should leave space at the end of every supervisory session to allow for discussion of what is working well in supervision, and what may be working less well” did not achieve consensus in the first round (72%) but did reach consensus in the second round (86%). The qualitative comments on this statement indicated that although no participants disagreed with the principle of seeking feedback from supervisees, some queried whether it is necessary to do so after every session, particularly where supervision takes place very regularly (e.g., weekly).

The statement “In emergency contexts, it is essential to make concessions on how supervision sessions are conducted, including the need for a private space, confidentiality and regularity” did not reach consensus in the first round (66%). In the qualitative comments for this statement, several participants commented that while flexibility is important in emergency settings, some aspects of supervision, such as confidentiality, should not be compromised. The statement was modified accordingly for the second round to read “In emergency contexts, it is essential to make concessions on how supervision sessions are conducted, but not on core aspects such as confidentiality.” Worded this way, the statement achieved consensus, with 82% agreement.

A few statements failed to reach consensus in either round. The statement “The skills needed to be a good supervisor, such as empathy, unconditional positive regard, and being a good active listener can all be taught” scored 57% consensus in the first round. The statement was modified in the second round to express the view that such qualities could be “developed upon” in training, and agreement rose to 73%, just short of the threhold for consensus.

The statement “It is important that peer supervision does not take place with less experienced supervisees, as they run the risk of communicating misinformation” failed to reach consensus (54%) in the first round. The qualitative comments on this statement indicated some confusion in relation to the wording of the statement, and so the statement was updated in the second round to read “Peer supervision should only take place between experienced professionals to avoid the risk of misinformation being communicated.” However, consensus on this item only increased to 57% in the second round, with several participants expressing the view that there are significant benefits to be harnessed through peer supervision, regardless of how experienced the supervisees are. One participant commented:

*We will seldom find a completely homogenous group. Some moderation of peer supervision is an advantage*.

Another statement that failed to reach consensus was “Monitoring and evaluation tools, such as feedback forms, are the best way to ensure quality supervision in MHPSS and protection programming and supervisee progress.” This statement scored 60% in the first round, which dropped to 51% in the second. Several participants felt that while feedback forms can be useful for some purposes, they are not necessarily the best way of evaluating the success of supervision. Alternative possibilities for fulfilling this goal suggested by participants included monitoring and evaluation processes outside of supervision, and tracking client outcomes. Others preferred verbal reports to elicit more ‘in-depth’ feedback than that obtained using structured forms. One participant noted that although anonymous feedback is ideal, true anonymity is often difficult to achieve in practice, particularly where teams are small.

The statement “Supervision can only be considered supervision when taking place in specially designated spaces, such as an office or meeting room” achieved very low consensus in the first round (18%). The qualitative comments for this round appeared to indicate that participants felt that a requirement to meet in an office or meeting room did not reflect the reality of humanitarian settings. The wording of the statement was changed in the second round to read “Supervision can only be considered supervision when taking place in specially designated spaces, such as an office, meeting room or other private space.” However, the statement achieved just 16% consensus in the second round.

The statement “It is possible for remote supervision to be as successful as face-to-face supervision” did not reach consensus (72% dropping to 68% in the second round), despite a modification in the second round that read “With some additional effort, it is possible for remote supervision to be as successful as face-to-face supervision.” Finally, the statement “A key role of the supervisor is to give advice about how to enhance the quality of MHPSS service delivery” did not reach consensus in the first round (49%), with comments expressing disagreement with the idea that the supervisor’s role is to impart information, rather than to guide the supervisee more collaboratively in their skill development. However, it also did not reach consensus in its modified form in the second round (51%) which read, “A key role of the supervisor is to guide supervisees’ discovery in relation to how to enhance the quality of MHPSS service delivery.”

## Gender analyses

The bivariate analyses of gender differences in responding suggested that three statements generated significantly different responses between men and women. For the statement that read “The issue of power dynamics between supervisor and supervisee is best addressed directly in supervision, by openly discussing sources of power and privilege that may be held by the supervisor and/or supervisee and how these might influence the supervisory relationship,” male gender was associated with slightly higher agreement (*t* (34) = -2.15, *p* = 0.04). In the qualitative comments for this item, one male participant strongly endorsed the statement:

*[Addressing power dynamics in this way] would help both parties to understand power dynamics and how they impact our roles in quality service delivery.*

However, a female respondent who selected the response ‘neither agree nor disagree’ wrote the following comment:

*The issue of power dynamics is very important; however it’s important to consider that in some cases this may not be feasible to discuss openly during supervision. It is vital that supervisors are aware of their own power dynamics and how this may influence the sessions with their staff. Some staff may not feel comfortable speaking with their supervisors openly about this, especially when the clinical supervisor and manager are the same role.*

Similarly, male gender was associated with slightly higher agreement with the statements “With some additional effort, it is possible for remote supervision to be as successful as face-to-face supervision” (*t* (28) = -2.23, *p* = 0.03) and “A key role of the supervisor is to guide supervisees’ discovery in relation to how to enhance the quality of MHPSS service delivery” (*t* (29) = −2.45, *p* = 0.02).

In relation to remote supervision, several reservations to this statement centred around the lack of evidence currently available on the subject. One female participant endorsed the statement with the following caveat:

*Research [studies] are still ongoing, but it also depends on the supervisor and does he/she feel confident to do it remotely.*

Another participant (female) stated:

*We have no evidence on this insofar as I am aware. I do think that in general, face-to-face meetings help in building a rapport and understanding the working context. However, remote supervision may have the benefit of feeling safe in having someone “outside the situation” to talk to.*

Finally, in relation to the statement about the function of supervision to enhance the quality of service provision, this statement was modified from the first round, where the statement first read that a key role of the supervisor was to “give advice” to the supervisee about enhancing the quality of services. Several participants highlighted that the wording relating to “giving advice” did not seem to embody the collaborative approach required for supportive supervision. For the second round, the wording “guiding supervisees’ discovery” was used instead, at the suggestion of one of the respondents. However, the newly worded item appeared to create some ambiguity about the meaning of the statement, resulting in a final consensus score of 51%.

## Discussion

This Delphi study aimed to examine the degree of consensus or disagreement among MHPSS stakeholders on 28 statements relating to the supervision of MHPSS interventions in humanitarian settings, with a view to developing a new set of guidelines for its delivery, entitled the Integrated Model for Supervision (IMS). Consensus and disagreement were examined both quantitatively (through consensus scores) and qualitatively (by analysing qualitative comments in support of participants’ votes). Potential gender differences in responses to each statement were also examined.

There was a high degree of consensus on most statements, including universal or near-universal agreement in relation to the use of active listening techniques in supervision, supervisors having access to their own supervision, and in relation to the use of Socratic questioning to guide supervisees’ critical reflection on their practice (consensus scores 97–100%). Accordingly, those statements which reached consensus (75% agreement or above) will be included in the IMS as guidance for best practice.

For the statements that did not reach consensus, the IMS guidelines will reflect the diversity of views and approaches to the issues in question. For example, the statement relating to remote supervision being as effective as face-to-face supervision did not reach consensus. Although guidance on remote supervision is important to include in the IMS, given the access difficulties and security issues often associated with working in humanitarian contexts (e.g. van der Veer et al. [24]), the guidance will acknowledge that face-to-face supervision, where possible, is preferable.

The findings in relation to peer supervision suggest that respondents did not agree that peer supervision should only be limited to more experienced professionals. This is consistent with other research suggesting unique benefits to be harnessed by this modality, such as reducing the hierarchical power disparity that exists between supervisors and supervisees of different experience levels [13]. The IMS will therefore recommend that peer supervision be made available to all practitioners, but that for less experienced practitioners, this support is complementary to individualised support from a more experienced practitioner. The IMS will also propose a system of moderation for peer supervision with less experienced practitioners, whereby a more experienced supervisor may make themselves available for when additional support or information is required.

The finding in relation to certain qualities of supervisors that are perceived as being largely inherent to certain personality types, and therefore needing to be screened for in prospective supervisors, is consistent with the findings of a previous study [17], which interviewed key informants about supervision of MHPSS. The study by Perera et al. [17] suggested that individuals with certain personal attributes should be selected to take on supervisory roles, rather than the role simply falling to individuals on the basis of reaching a certain level of seniority. Based on these findings, the IMS will provide guidance on the types of personal qualities and attributes that it may be useful to screen for when appointing MHPSS supervisors and will provide some suggestions around how to do so.

Some of the items that did reach consensus will also require somewhat nuanced discussion in the guidance. For example, the statement relating to the discussion of personal issues in supervision did not reach consensus in the first round, but it did reach consensus in the second round, following discussion in the qualitative comments about some limits to the nature of such discussions. Several comments noted that while supervision should include discussion of how personal issues are affecting the supervisee’s work, it is not the role of supervision to *resolve* such issues. However, several participants noted that supervisors should assist the supervisee in finding the right supports to address their issues, where these fall outside the remit of supervisory discussions.

Few gender differences in responses were identified. However, the bivariate analyses suggested small differences in responding in relation to three statements. Women had slightly lower levels of agreement with the statement that expressed the view that power dynamics should be addressed directly within supervision sessions. Some of the qualitative comments suggest that women respondents may have been somewhat more likely to feel that conversations about power dynamics could be experienced as uncomfortable by supervisees. It may be the case that women are more likely to have experienced such discomfort themselves in supervisory contexts. These reservations highlight a need to ensure that such conversations are supportive and respectful of the trauma-informed principles of empowerment and collaboration [22], and not imposed on supervisees in a way that causes anxiety. Research on multicultural supervision settings has suggested that supervisors creating space for supportive dialogue about diversity has the potential to enhance supervisees’ self-efficacy and satisfaction with the supervisory process [14, 18]. Satisfaction with the supervisory process, in turn, has been associated with better mental health and occupational outcomes among emergency responders [4]. Therefore, it seems that developing more detailed guidance on how to approach such conversations would be a useful avenue for future investigations.

## Limitations

This study sampled a diverse spectrum of MHPSS stakeholders, and generated data in relation to their agreement or disagreement on key principles on supervision of MHPSS in humanitarian settings. However, several limitations should be noted. Minimal information in relation to participants’ level of experience was collected - gathering data on participants’ years of experience working in humanitarian settings, for example, would have been beneficial. The sampling procedure entailed self-selection bias. The overall sample size was relatively small, and the drop-out rate high, which is particularly important to bear in mind when interpreting the bivariate analyses. Significant gender differences in responses were identified in relation to just three statements. However, these associations should be interpreted with caution. Conducting multiple comparisons presents the possibility of Type 1 error. It is also possible that some associations were missed due to low statistical power. Additionally, the sample included a disproportionate number of women respondents, which may have introduced a degree of bias into the findings overall. It was not possible within the time constraints of the project to conduct a pilot study. Doing so may have enhanced the clarity and accessibility of the statements presented. This study is also affected by the limitations inherent in the use of cutoff scores; the selection of a 75% threshold for consensus necessitates accepting the validity of statements that achieve 75% consensus, but not 74% consensus, when, in reality, this may represent very little meaningful difference in terms of participants’ level of agreement with a given statement.

## Conclusions

This study provides important information for the development of new guidance on the supervision of MHPSS in humanitarian emergency settings, offering unique insights in relation to supervision in emergencies, and highlighting the balance between best practice and the realities of operating in highly challenging contexts. However, the findings indicate several core elements of MHPSS supervision that must not be compromised (e.g., providing a confidential space for supervision to take place) even in challenging circumstances. Analyses of gender differences suggested potential differing opinions and approaches to supervision between genders, although it is possible that the small sample size may have obscured other differences that were present. Further examination of gender differences in approaches to MHPSS supervision may be a useful priority for future research, as well as consideration of how views and approaches may differ between MHPSS workers of varying experience levels and across diverse contexts.

## Data Availability

The data supporting this study contains potentially identifiable information and is not publicly available for data protection reasons.

## Abbreviations

TCGH: Trinity Centre for Global Health
IFRC: International Federation of Red Cross and Red Crescent Societies
MHPSS: Mental health and psychosocial support
IMS: Integrated Model for Supervision
IASC: Inter-Agency Standing Committee
WHO: World Health Organization
PEPFAR: U.S. President’s Emergency Plan for AIDS Relief
UNAIDS: The Joint United Nations Programme on HIV and AIDS
SAMHSA: Substance Abuse and Mental Health Services Administration

## References

[1] Ager A, Pasha E, Yu G, Duke T, Eriksson C, Cardozo BL (2012). Stress, mental health, and burnout in national humanitarian aid workers in Gulu, Northern Uganda. J Trauma Stress.

[2] Aldamman K, Tamrakar T, Dinesen C, Wiedemann N, Murphy J, Hansen M, Vallières F (2019). Caring for the mental health of humanitarian volunteers in traumatic contexts: the importance of organisational support. Eur J Psychotraumatol.

[3] Barrett D, Heale R (2020). What are Delphi studies?. Evid Based Nurs.

[4] Brooks SK, Dunn R, Amlôt R, Greenberg N, Rubin GJ (2016). Social and occupational factors associated with psychological distress and disorder among disaster responders: a systematic review. BMC Psychol.

[5] Cardozo BL, Crawford CG, Eriksson C, Zhu J, Sabin M, Ager A, Simon W (2012). Psychological distress, depression, anxiety, and burnout among international humanitarian aid workers: a longitudinal study. PLoS ONE.

[6] Charlson F, van Ommeren M, Flaxman A, Cornett J, Whiteford H, Saxena S (2019). New WHO prevalence estimates of mental disorders in conflict settings: a systematic review and meta-analysis. Lancet.

[7] Connorton E, Perry MJ, Hemenway D, Miller M (2012). Humanitarian relief workers and trauma-related mental illness. Epidemiol Rev.

[8] Diamond IR, Grant RC, Feldman BM, Pencharz PB, Ling SC, Moore AM, Wales PW (2014). Defining consensus: a systematic review recommends methodologic criteria for reporting of Delphi studies. J Clin Epidemiol.

[9] Helmer-Hirschberg O (1967). Analysis of the Future: The Delphi Method.

[10] Hensel JM, Ruiz C, Finney C, Dewa CS (2015). Meta-analysis of risk factors for secondary traumatic stress in therapeutic work with trauma victims. J Trauma Stress.

[11] Inter-Agency Standing Committee (IASC) (2007). IASC Guidelines on Mental Health and Psychosocial Support in Emergency Settings.

[12] International Federation of Red Cross and Red Crescent Societies (IFRC). Trinity Centre for Global Health (TCGH) & Anglemap (2020). *Supervision: The Missing Link Research Synthesis from Three Remote Workshops*. https://pscentre.org/?resource=supervision-the-missing-link.

[13] Kemp CG, Petersen I, Bhana A, Rao D (2019). Supervision of task-shared mental health care in low-resource settings: a commentary on programmatic experience. Global Health.

[14] Marquez L, Kean L. *Making Supervision Supportive and Sustainable: New Approaches to Old Problems.* Maximising Access and Quality Paper No. 4;2002. https://pdf.usaid.gov/pdf_docs/PNACS924.pdf.

[15] McBride K, Bitanihirwe B, Vallières F, Perera C, Wiedemann N, Blum TP. *Supervision for the delivery of Mental Health and Psychosocial Support in Emergency Humanitarian Settings: A desk review report; 2020*. https://app.mhpss.net/?get=294/supervision-the-missing-link-desk-review-may-2020-1.pdf.

[16] Ng KM, Smith SD (2012). Training level, acculturation, role ambiguity, and multicultural discussions in training and supervising international counseling students in the United States. Int J Adv Couns.

[17] Perera C, McBride K, Travers Á, Tingsted Blum P, Wiedemann N, Dinesen C, Bitanihirwe B, Vallières F (2021). Towards an integrated model for supervision for mental health and psychosocial support in humanitarian emergencies: a qualitative study of practitioners’ perspectives. PLoS ONE.

[18] Schultz T, Baraka MK, Watson T, Yoo H. How do ethics translate? Identifying ethical challenges in transnational supervision settings. Int J Adv Counsel. 2019. 10.1007/s10447-019-09388-4.

[19] Shah SA, Garland E, Katz C (2007). Secondary traumatic stress: Prevalence in humanitarian aid workers in India. Traumatology.

[20] Strohmeier H, Panter-Brick C (2020). Living with transience in high‐risk humanitarian spaces: Gendered experiences of international staff and policy implications for building resilience. Disasters.

[21] Strohmeier H, Scholte WF, Ager A (2018). Factors associated with common mental health problems of humanitarian workers in South Sudan. PLoS ONE..

[22] Substance Abuse and Mental Health Services Administration. *SAMHSA’s Concept of Trauma and Guidance for a Trauma-Informed Approach*. HHS Publication No. (SMA) 14-4884. Rockville, MD: Substance Abuse and Mental Health Services Administration. 2014. https://ncsacw.samhsa.gov/userfiles/files/SAMHSA_Trauma.pdf.

[23] Tol WA, Ager A, Bizouerne C, Bryant R, El Chammay R, Colebunders R, van Ommeren M (2020). Improving mental health and psychosocial wellbeing in humanitarian settings: reflections on research funded through R2HC. Conflict Health.

[24] van der Veer G, de Jong K, Lansen J (2004). Clinical supervision for counsellors in areas of armed conflict. Intervention..

[25] WHO, PEPFAR & UNAIDS‎. *Task shifting: rational redistribution of tasks among health workforce teams: global recommendations and guidelines*. Geneva: World Health Organization. 2007. https://apps.who.int/iris/handle/10665/43821.

[26] World Health Organization. *Training for mid-level managers (MLM)*. *Module 4: Supportive supervision*. 2008. www.who.int/vaccines-documents/.

